# A longitudinal causal graph analysis investigating modifiable risk factors and obesity in a European cohort of children and adolescents

**DOI:** 10.1038/s41598-024-56721-y

**Published:** 2024-03-21

**Authors:** Ronja Foraita, Janine Witte, Claudia Börnhorst, Wencke Gwozdz, Valeria Pala, Lauren Lissner, Fabio Lauria, Lucia A. Reisch, Dénes Molnár, Stefaan De Henauw, Luis Moreno, Toomas Veidebaum, Michael Tornaritis, Iris Pigeot, Vanessa Didelez

**Affiliations:** 1 Leibniz Institute for Prevention Research and Epidemiology - BIPS, Achterstr. 30, 28359 Bremen, Germany; 2 Faculty of Mathematics and Computer Science, University of Bremen, Bremen, Germany; 3 Department of Consumer Research, Communication and Food Sociology, Justus-Liebig-University, Gießen, Germany; 4 Department of Management, Society and Communication, Copenhagen Business School, Frederiksberg, Denmark; 5 Epidemiology and Prevention Unit, Fondazione IRCCS Istituto Nazionale dei Tumori di Milano, Milan, Italy; 6 School of Public Health and Community Medicine, Institute of Medicine, Sahlgrenska Academy, University of Gothenburg, Gothenburg, Sweden; 7 Institute of Food Sciences, CNR, Avellino, Italy; 8 El-Erian Institute of Behavioural Economics and Policy, University of Cambridge, Cambridge, UK; 9 Department of Paediatrics, Medical School, University of Pécs, Pécs, Hungary; 10 Department of Public Health and Primary Care, Faculty of Medicine and Health Sciences, Ghent University, Ghent, Belgium; 11 GENUD (Growth, Exercise, Nutrition and Development) Research Group, Instituto Agroalimentario de Aragón (IA2), Instituto de Investigación Sanitaria de Aragón (IIS Aragón), University of Zaragoza, Zaragoza, Spain; 12 Department of Chronic Diseases, National Institute for Health Development, Tallinn, Estonia; 13 Research and Education Institute for Child Health, Strovolos, Cyprus

**Keywords:** Audio-visual media consumption, Causal structure learning, DAG, Healthy diet, IDEFICS/I.Family cohort, Multiple imputation, PC-algorithm, Physical activity, Sleep, Well-being, Obesity, Risk factors, Statistics

## Abstract

Childhood obesity is a complex disorder that appears to be influenced by an interacting system of many factors. Taking this complexity into account, we aim to investigate the causal structure underlying childhood obesity. Our focus is on identifying potential early, direct or indirect, causes of obesity which may be promising targets for prevention strategies. Using a causal discovery algorithm, we estimate a cohort causal graph (CCG) over the life course from childhood to adolescence. We adapt a popular method, the so-called PC-algorithm, to deal with missing values by multiple imputation, with mixed discrete and continuous variables, and that takes background knowledge such as the time-structure of cohort data into account. The algorithm is then applied to learn the causal structure among 51 variables including obesity, early life factors, diet, lifestyle, insulin resistance, puberty stage and cultural background of 5112 children from the European IDEFICS/I.Family cohort across three waves (2007–2014). The robustness of the learned causal structure is addressed in a series of alternative and sensitivity analyses; in particular, we use bootstrap resamples to assess the stability of aspects of the learned CCG. Our results suggest some but only indirect possible causal paths from early modifiable risk factors, such as audio-visual media consumption and physical activity, to obesity (measured by age- and sex-adjusted BMI z-scores) 6 years later.

## Introduction

Childhood obesity is a serious public health problem in many countries^1^ leading to severe co-morbidities in later life such as type 2 diabetes, cardiovascular diseases, certain types of cancer, depression and other psychosocial problems^2–4^. Prevention of obesity in children and adolescents seems to be the “only feasible solution” to tackle the obesity epidemic^5^. But prevention strategies need promising targets to achieve any public health effect. However, childhood obesity is a complex disorder that appears to be influenced by an interacting system of individual behaviour, group and societal settings such as family, school or the country-specific infrastructure (e.g. public health system, built environment)^6^.

While most investigations focus on single exposure-outcome associations, our approach is to assess the complex interplay of obesity-related factors over the transition from childhood to adolescence by estimating a “cohort causal graph” (CCG), i.e. a causal graph that allows for the longitudinal structure of cohort data, including early life, individual, familial and social aspects using data from the European IDEFICS/I.Family cohort^7^. Causal discovery is still rarely used in medicine^8^, epidemiology^9,10^, with the most of them in the field of genetics^11,12^. This might be because the available methodologies and available software were previously limited to handling simple data structures. For the first time, our analysis infers a causal graph from observational data in which we simultaneously account for the temporal order of the covariates^13,14^, mixed variable scales and missing values^11^. The main aim is to identify plausible causal paths from early modifiable risk factors, such as diet, physical activity (PA), media consumption, subjective well-being and sleep, to body mass index (BMI) 6 years later. These may suggest or rule out potential targets for future obesity prevention strategies.

## Methods

### Study population

The IDEFICS/I.Family cohort^7,15^ is a European cohort study initiated with the overall aims to identify and prevent dietary and lifestyle induced health effects in infants, children and adolescents. The baseline survey (B) was conducted in 2007/08 in eight European countries (Belgium, Cyprus, Estonia, Germany, Hungary, Italy, Spain and Sweden) with 16,229 participating children (2 to 9.9 years old). The first follow-up examinations (FU1, conducted in 2009/10) included 13,596 children and applied the same standardised assessments. The second follow-up examinations (FU2, conducted in 2013/14) enrolled 7105 children who already participated at B or FU1.

Ethical approval was obtained from the responsible ethics committees in each country and all research was performed in accordance with the Declaration of Helsinki principles (Belgium: Ethics Committee of the University Hospital Ghent (EC UZG 2007/243, B670201316342); Cyprus: National Bioethics Committee (EEBK/EM/2007/16, EEBK/ETI/2012/33); Estonia: Tallinn Medical Research Ethics Committee of the National Institutes for Health Development (1093, 128); Germany: Ethics Committee of the University Bremen (16/01/2007, 11/12/2012); Hungary: Scientific and Research Ethics Committee of the Medical Research Council Budapest (22-156/2007-1018EKU, 4536/2013/EKU); Italy: Ethics Committee of the Health Office Avellino (2/CE, 12/12); Spain: Ethics Committee for Clinical Research of Aragon (PI07/13, PI13/0012); Sweden: Regional Ethical Review Board of Gothenburg (264-07, 927-12). All children and their parents provided oral and written informed consent, respectively, before examinations and/or the collection of samples, subsequent analysis and storage of personal data and collected samples. Teens older than 12 years were asked to provide their written consent using a simplified version of the consent form. Study subjects and their parents could opt out of each single study component.

### Covariates

We included variables reflecting eating behaviour, lifestyle, social, cultural and environmental factors that are assumed to be related to overweight and obesity across the early life course. A detailed description of all measurements and their units used in our analysis is provided in Table 1 and in the supplement. Some of these variables are time-invariant and would not be targeted by any intervention programme in later childhood, such as region of residence or migration background. Other time-invariant variables might impact a child’s development during pregnancy and as an infant, such as mother’s age at birth or breastfeeding duration; we will refer to these as early life factors. All other variables are time-varying and were measured repeatedly. Age- and sex-specific BMI z-scores (BMI) for children and adolescents were calculated according to the extended IOTF criteria^16^; for simplicity we refer to these as BMI. Mother’s BMI was assessed at each survey in kg/m^2^. The homeostatic model assessment (HOMA-IR, short HOMA) index^17^ served as a marker for insulin resistance. The diet of the child was measured by a validated FFQ^18^ and was classified by an adapted version of the Youth Healthy Eating Index (YHEI)^19^. The YHEI assesses the consumption frequencies of both healthy and unhealthy food as well as eating behaviours, where a higher score indicates a healthier diet^20^. PA was measured by questionnaire, and an audio-visual media consumption score (AVM) was used as proxy for sedentary behaviour. Total sleep duration including nocturnal sleep was estimated based on 24-h dietary recall data at baseline^21^ and quantified by self-reports at the two follow-ups. Multiple dimensions of psychosocial well-being were assessed by questionnaire which was developed for parents’ response on behalf of children and adolescents^22^. Children above the age of 12 completed the questionnaires for themselves. Further details on the study population and used covariates are given in the supplement.

**Table 1:** Variables used in the analysis with units and further explanations.

Tiers	Variable/node	Unit	Comments
Context	Sex	Female, male	Sex of child
Context	Region	North, Central, South	Place of residence in one of the following European countries: North (Estonia, Sweden), Central (Belgium, Germany, Hungary), South (Cyprus, Spain)
Context	Migrant	No, yes	Children were assumed to have a migrant background if they usually speak with their parents in a language other than the national language of the corresponding country
Early life	Mother's age at birth	*Years*	
Early life	Total breastfeeding	*Months*	Months of breastfeeding, also in combination with other food, prior child's diet was fully integrated into usual household diet
Early life	Birthweight	*Gram*	
Early life	Weeks of pregnancy	*Weeks*	
Early life	Formula milk	No, yes	Type of feeding prior child's diet was fully integrated into the usual household diet
Early life	HH diet	*Months*	Month when the child was introduced into the household's diet
Early life	Smoking during pregnancy	No, yes	Mother consumed tobacco during pregnancy
B, FU1, FU2	Age	*Months*	
B, FU1	School	Kindergarten, school, neither one	Child attended kindergarten/pre-school, school or neither one
B, FU1, FU2	AVM	*h/day*	Audio-visual media consumption score: average hours per day spent with TV, videos, or DVDs, accounting for weekdays and weekends. Hours using the internet per week were only assessed at FU2
B, FU1, FU2	zBMI	*z-score*	Z-scores of the body mass index (kg/m^2^). Body weight was measured in fasting state in light underwear on a calibrated scale accurate to 0.1 kg (adapted Tanita BC 420 MA for children ≤ 6 years, Tanita BC 418 MA for children > 6 years, Tanita Europe GmbH, Sindelfingen, Germany); height was measured to the nearest 0.1 cm by a SECA 225 Stadiometer (Seca GmbH & Co. KG., Hamburg, Germany)
B, FU1, FU2	Mother's BMI	*kg/m*^*2*^	Body mass index of the mother, derived from self-reported weight and height
B, FU1, FU2	Daily family meals	No, yes	The family has a meal together at least once a day
B, FU1, FU2	Income	Low, middle, high	Country-specific household income categories, harmonised between countries^60^
B, FU1, FU2	ISCED	Low, middle, high	International Standard Classification of Education: The partners' highest attained level of education^61^
B, FU1, FU2	PA	*h/day*	Physical activity measured by questionnaire based on the reported average time spent playing outdoors (hours/week) and the time being in recreation areas or doing sports in a sport club (hours/week)
B, FU1, FU2	Sleep	*h/day*	Nocturnal sleep in hours was assessed by self-reports in FU1 and FU2. The average nocturnal sleep (hours/night) was calculated as the weighted average of reported usual weekday and weekend sleeping times. At baseline, nocturnal sleep was derived based on 24-h dietary recall data where the parents were asked ‘What time did your child go to bed?’ and ‘What time did your child get up?’
B, FU1, FU2	Well-being	*%*	Composite sum score; it sums up the answers of 16 items reporting emotional well-being, self-esteem, family relations and peer contacts during the last week, where each item ranges from 0 to 3 points^22,62^
B, FU1, FU2	YHEI	*%*	Youth healthy eating score^20^
B, FU1, FU2	HOMA	*z-score*	Z-score of the HOmeostatic Model Assessment index to quantify insulin resistance; the HOMA-IR index [pg/ml*mg/dl] was calculated from insulin and glucose obtained from blood samples
FU2	Alcohol	No, yes	Ever alcohol drinking in teen's life-time
FU2	Puberty	Pre- or early pubertal, pubertal	Pubertal status based on development of voice (boys) and menarche (girls)^63^. Different pubertal stages were displayed in the questionnaire to assist the self-assessment
FU2	Smoking	No, yes	Ever smoking tobacco in teen's life-time

Background knowledge was used to order them into different tiers. Units of continuous variables are given in italics.

*B* baseline, *FU1* first follow-up, *FU2* second follow-up.

### Statistical analysis

For our analyses, only children who participated in all three surveys were considered. Multiple imputation (MI) was applied to avoid loss of study subjects and to reduce potential bias due to missing values^23^; specifically we used tenfold imputation with random forests as implemented in the R-package *mice*^24^. MI assumes that values were missing at random (MAR). To strengthen the plausibility of the MAR assumption, the imputation models were fitted on a larger dataset containing additional variables that contribute to the various scores such as AVM or well-being^23^.

To estimate the cohort causal graph (CCG), we applied a method of causal discovery known as PC-algorithm^25,26^. The algorithm outputs empirically plausible causal directed acyclic graphs (causal DAGs) suggesting direct and indirect causal relations, as shown by directed edges or directed paths. We chose this particular algorithm because other, especially likelihood-based approaches typically make more implicit or explicit distributional assumptions which would seem highly implausible for the given cohort data. While the PC-algorithm also makes assumptions, there is some more robustness of our approach, e.g. in the context of multiple imputation^27^. As a DAG represents certain conditional (in)dependencies between variables^28^, the PC-algorithm proceeds by investigating conditional independencies in the data using statistical tests, and then determines all DAGs that agree with these independencies. The result is not unique since different DAGs can represent the same conditional independencies, i.e. certain causal structures are indistinguishable. Instead, the algorithm outputs the equivalence class of all DAGs that represent the detected conditional independencies. This class is represented by a so-called completed partially directed acyclic graph (CPDAG)^29^ containing directed and undirected edges, where an undirected edge means that both causal directions occur in the equivalence class. The validity of the PC-algorithm relies on the assumptions of causal sufficiency, i.e. absence of latent confounding, and of faithfulness, under which the PC-algorithm consistently selects the true CPDAG^25^. Of note, while the causal interpretation of directed edges or paths in the output of causal discovery algorithms relies on causal sufficiency, which may often be implausible, the absence of such edges and paths can still be interpreted as absence of causal relations even without causal sufficiency.

The PC-algorithm had to be modified for application to multiply imputed cohort data^11,27,30^. Further, to account for the cohort structure we used the tiered PC-algorithm *tPC*^31^. This was then combined with functions from *micd*^32^ to deal with multiply imputed data containing a mix of categorical and continuous variables. The R packages *micd* and *tPC* are both extensions of *pcalg*^33^. The *tPC*-algorithm outputs a maximally oriented partially directed acyclic graph (MPDAG), which is similar to a CPDAG but can contain more directed edges due to background knowledge^13,34^. *tPC* determines an MPDAG under the restriction that edges are prohibited from pointing backwards in time which also reduces the number of required statistical tests for conditional independencies. In our analysis we pre-specified the following ordering: region, sex and migration → ISCED and income at baseline → all early life factors → baseline variables → ISCED and income at FU1 → remaining FU1 variables → ISCED and income at FU2 → remaining FU2 variables. Additionally, specific orientations between certain pairs of variables were prohibited, for example from breastfeeding to birth weight. We carried out a number of alternative and sensitivity analyses to check the robustness of the estimated MPDAG against specific analytical choices: (a) while the main analysis used a nominal level of 0.05 for the conditional independence tests, we compared this with a nominal level of 0.1 (MI-0.1); (b) using test-wise deletion (TWD) instead of MI and (c) applying a different, likelihood-based, causal discovery algorithm which uses the EM algorithm for missing values^35^. Moreover, to assess the general stability of the output we drew 100 bootstrap samples from the analysis data, applied to each a single random forest imputation using the same imputation model as in the main analysis, and then estimated 100 bootstrap graphs (BGs). Thus, we can take the frequencies of interesting causal structures in the bootstrap samples as indication of their stability, e.g. specific edges (direct causal links) or indirect links via (partially) directed paths between exposures and outcome. In a directed path, all edges between two nodes are directed, while in a partially directed path, at least one edge between two nodes is undirected. More background on causal graphs and other graph characteristics are described in the supplement.

## Results

### Study sample

The study sample included 5,112 children who participated in all three surveys. Table 2 shows that children were on average aged 5.9 years at baseline and 11.7 years at FU2. At baseline, 12.6% of the children have overweight and 6.7% suffer from obesity. BMI z-scores increased on average by approx. 0.2 standard deviations (SD) over the years (0.32 to 0.55). The overall number of missing values was 15% with some variables exhibiting very large numbers of missings such as PA at FU2 (50.1%) (see Figure S1 and Table S1 characteristics after imputation). Diagnostic plots of the multiply imputed data were satisfactory (see Figure S2).

**Table 2:** Characteristics of children in the IDEFICS/I.Family cohort participating in all three surveys from 2007 to 2014.

Time-invariant variables	N = 5112^a^		
Region			
Central (Belgium, Germany, Hungary)	1378 (27%)		
North (Estonia, Sweden)	1475 (29%)		
South (Cyprus, Italy, Spain)	2259 (44%)		
Female	2505 (49%)		
Migration background	319 (6.7%)		
Missing	385		
Completed weeks of pregnancy	39.08 (1.88)		
Missing	2995		
Tobacco smoking during pregnancy			
Never	4285 (88.7%)		
Rarely	171 (3.5%)		
Several occasions a week	150 (3.1%)		
Daily	226 (4.7%)		
Missing	280		
Mother's age at birth (yrs)	29.8 (5.0)		
Missing	494		
Birthweight (g)	3345 (574)		
Missing	180		
Total breastfeeding (months)	6.8 (6.3)		
Missing	247		
Was fed with formula milk	2640 (51.6%)		
Missing	0		
Fully integrated into household's diet (month)	14.5 (6.5)		
Missing	722		

Time-varying variables	Baseline, N = 5112^a^	FU1, N = 5112^a^	FU2, N = 5112^a^
Age [yrs]	5.89 (1.78)	7.87 (1.79)	11.69 (1.81)
School			
Kindergarten	2452 (51.7%)	1100 (23.4%)	–
School	2250 (47.4%)	3584 (76.4%)	–
Neither	41 (0.9%)	8 (0.2%)	–
Missing	369	420	–
BMI z-score	0.32 (1.17)	0.43 (1.17)	0.55 (1.11)
BMI			
Underweight	570 (11.2%)	506 (9.9%)	394 (7.7%)
Normal weight	3559 (69.6%)	3397 (66.5%)	3352 (65.6%)
Overweight	643 (12.6%)	819 (16.0%)	986 (19.3%)
Obesity	340 (6.7%)	390 (7.6%)	380 (7.4%)
Well-being (%)	84 (10)	82 (10)	82 (11)
Missing	636	552	625
Audio-visual media consumption (h/day)	1.57 (0.89)	1.89 (0.94)	2.94 (1.83)
Missing	306	394	654
Physical activity (h/week)	18 (11)	18 (10)	17 (9)
Missing	252	357	2561
Nocturnal sleep (h/day)	10.19 (0.96)	10.01 (0.90)	9.29 (1.03)
Missing	2130	781	449
Youth healthy eating index (%)	63 (11)	63 (11)	57 (11)
Missing	343	446	350
Daily family meals	3488 (73.5%)	3548 (76.5%)	2662 (67.1%)
Missing	367	476	1147
Homa index z-score	0.02 (1.10)	0.40 (0.97)	0.13 (1.15)
Missing	2902	2466	1911
Pubertal	–	–	1931 (41.2%)
Missing	–	–	423
Ever alcohol drinking	–	–	738 (32.7%)
Missing	–	–	2852
Ever tobacco smoking	–	–	213 (9.3%)
Missing	–	–	2812
Mother's BMI (kg/m^2^)	23.8 (4.2)	24.0 (4.3)	25.5 (5.1)
Missing	271	384	2732
Household's income			
Low	1612 (36.0%)	1,410 (31.4%)	1,197 (28.5%)
Middle	1179 (26.3%)	1,130 (25.2%)	1,451 (34.5%)
High	1693 (37.8%)	1,949 (43.4%)	1,559 (37.1%)
Missing	628	623	905
ISCED			
Low	254 (5.1%)	232 (4.8%)	248 (4.9%)
Middle	2,085 (42.2%)	2,004 (41.5%)	2,147 (42.3%)
High	2,600 (52.6%)	2,590 (53.7%)	2,681 (52.8%)
Missing	173	286	36

^a^n (%); mean (standard deviation).

*FU1* first follow-up, *FU2* second follow-up, *BMI* body mass index, *ISCED* highest parental education (International Standard Classification of Education).

### Cohort causal graph

**Figure 1:**
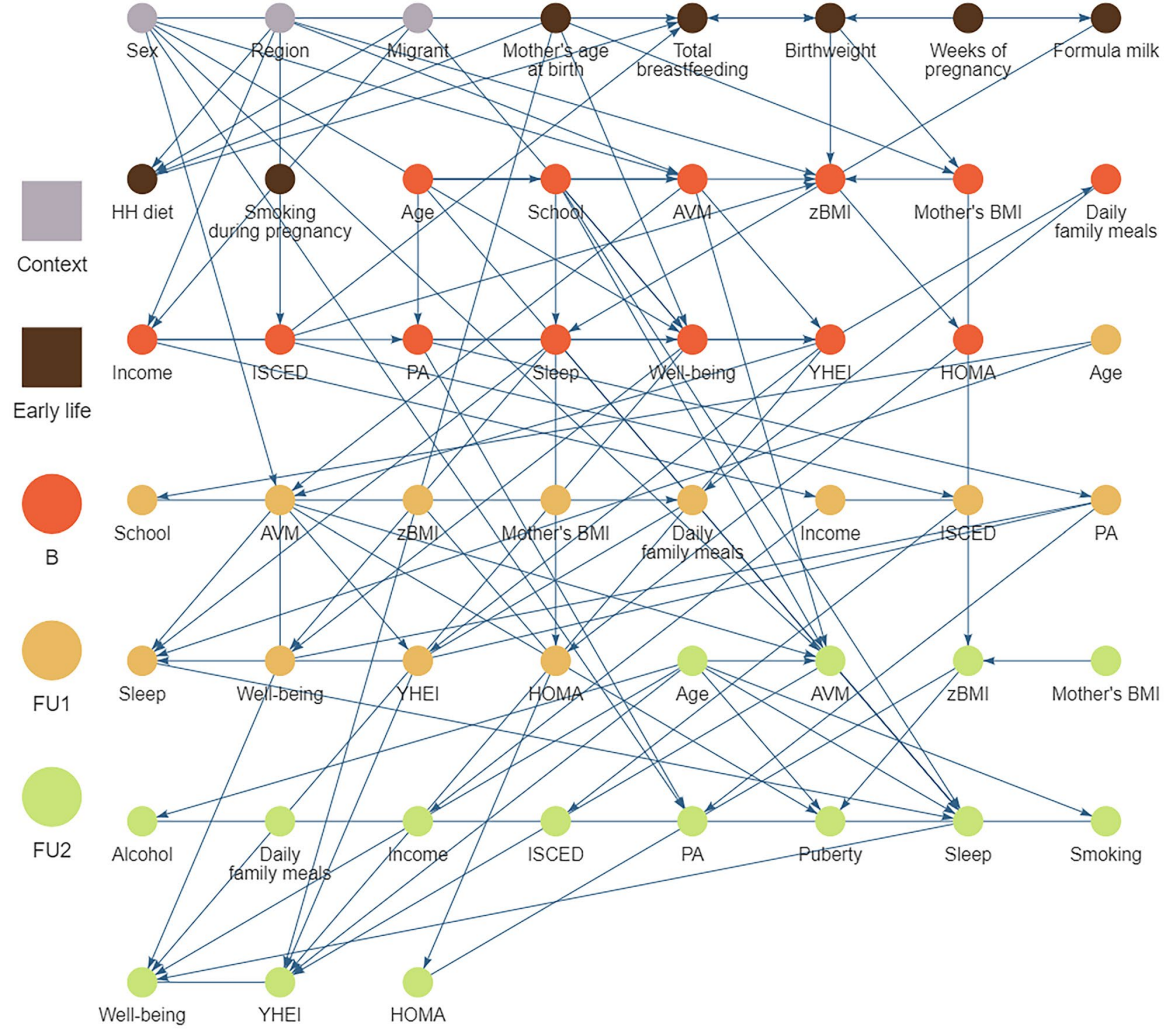
Causal graph of childhood obesity based on N = 5112 European children and adolescents born between 1997 and 2006 estimated by the tiered PC-algorithm for multiple imputed datasets. The nodes colours correspond to the different stages of the life course. Edges without arrowheads could not be orientated by the algorithm. An overlap of nodes and edges was unavoidable. We advise to look at the interactive graphs here: https://bips-hb.github.io/ccg-childhood-obesity/. *AVM* audio-visual media consumption, *B* baseline, *FU1* first follow-up, *FU2* second follow-up, *HH* diet: month when the child was introduced into the household's diet, *HOMA* homeostatic model assessment-insulin resistance, *ISCED* highest parental education (International Standard Classification of Education), *PA* physical activity, *YHEI* youth healthy eating index, *zBMI* body mass index z-score.

**Figure 2:**
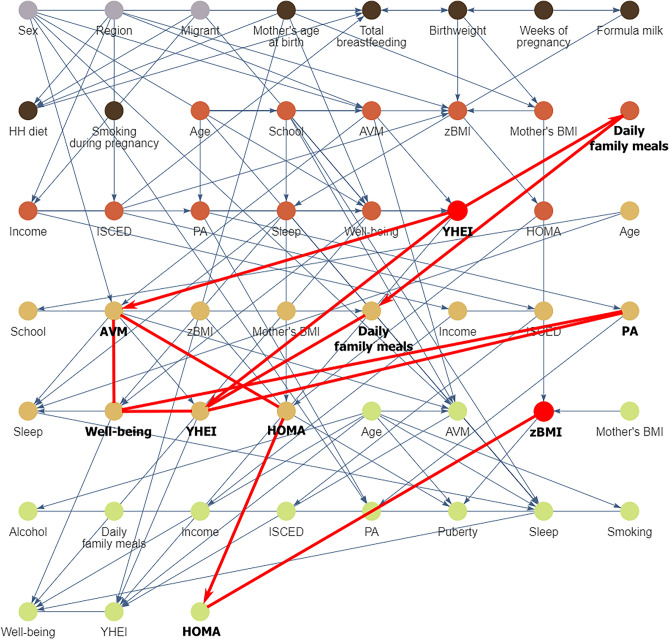
All five possible causal paths between the Youth Healthy Eating Index (YHEI) at baseline and zBMI at the second follow-up (*AVM* audio-visual media consumption, *PA* physical activity). *AVM* audio-visual media consumption, *B* baseline, *FU1* first follow-up, *FU2* second follow-up, *HH* *diet* month when the child was introduced into the household's diet, *HOMA* homeostatic model assessment-insulin resistance, *ISCED* highest parental education (International Standard Classification of Education), *PA* physical activity, *YHEI* youth healthy eating index, *zBMI* body mass index z-score.

The CCG resulting from our main analysis is shown in Fig. 1 (see also https://bips-hb.github.io/ccg-childhood-obesity for an interactive graph). Overall the graph had 104 edges linking 51 variables, of which 12 could not be oriented. Focusing on BMI as outcome, there were direct links from region, familial educational level, birthweight and mother’s BMI (B) to BMI (B); in contrast, there were no paths from any of the modifiable risk factors to BMI (B). However, all of these modifiable baseline factors (sleep, AVM, YHEI, PA, well-being) were possible ancestors and hence possible causes of BMI in both follow-ups (cf. Table 3), i.e. they had partially directed paths to BMI. These included paths from all five modifiable baseline risk factors to BMI six years later. For instance, there were five partially directed paths from YHEI (B) to BMI (FU2) (Fig. 2). Almost all paths between exposures and BMI (FU2) went through AVM (FU1) and HOMA (FU1, FU2), many also through well-being (FU1) and some through YHEI (B). In the CCG we also see that the exposures themselves were moderately interconnected within the same tier and across time, with many orientations of edges among the exposures at FU1 being undecidable. Note also that most repeated measurements were linked by edges with the exception of BMI.

**Table 3:** Possible ancestors of BMI at baseline, first and second follow up.

Tier	Ancestors of BMI (B)	Ancestors of BMI (FU1)	Ancestors of BMI (FU2)
C	Sex	Sex	Sex
C	Region	Region	Region
C	Migrant	Migrant	Migrant
ELF	Mother’s age at birth	Mother’s age at birth	Mother’s age at birth
ELF	Birthweight	Birthweight	Birthweight
ELF	Weeks of pregnancy	Weeks of pregnancy	Weeks of pregnancy
ELF		Formula milk	Formula milk
B	Income	Income	Income
B	ISCED	ISCED	ISCED
B	Mother’s BMI	Mother’s BMI	Mother’s BMI
B	Age	Age	Age
B		School	School
B		AVM	AVM
B		BMI	BMI
B		PA	PA
B		Sleep	Sleep
B		Well-being	Well-being
B		YHEI	YHEI
B		HOMA	HOMA
FU1	*f.p.*	AVM (FU1)	AVM (FU1)
FU1	*f.p.*		BMI (FU1)
FU1	*f.p.*	PA (FU1)	PA (FU1)
FU1	*f.p.*	Well-being (FU1)	Well-being (FU1)
FU1	*f.p.*	HOMA (FU1)	HOMA (FU1)
FU2	*f.p.*	*f.p.*	Mother’s BMI (FU2)
FU2	*f.p.*	*f.p.*	HOMA (FU2)

*f.p.*: Path between a pair of vertices was forbidden a priori (e.g. due to time constraints).

*AVM* audio-visual media consumption, *BMI* body mass index, *B* baseline, *C* context variables, *ELF* early life factors, *FU1* first follow-up, *FU2* second follow-up, *HOMA* homeostatic model assessment-insulin resistance, *ISCED* highest parental education (International Standard Classification of Education), *PA* physical activity, *sleep* nocturnal sleep, *YHEI* youth healthy eating index.

### Bootstrap analysis

We assessed the stability of selected features of the main CCG based on 100 BGs. Of the 104 edges in the main CCG, 36 were found in more than 80% of BGs, with a further six edges in more than 70% of BGs (see Table S2). Of these edges, 16 were between repeated measures of the same variable, e.g. HOMA.FU1-HOMA.FU2, and 13 emanated from modifiable risk factors. In contrast, 50 edges occurred in 50% or fewer of the BGs. The presence of any paths from exposures to BMI was rather stable. Specifically, we considered directed or partially directed paths from baseline modifiable exposures to later BMI (FU2) (see Table 4). The most frequent were paths from YHEI to BMI (84% of BGs), while paths from sleep duration to BMI were in 75% of the BGs; paths from the other three baseline exposures (well-being, AVM, PA) to BMI occurred in 80% of the BGs. There were mostly multiple causal paths found between an exposure and the outcome. For instance, the median number of different (partially) directed paths from AVM (B) to BMI (FU2) found in each BG was 20. No BGs ever contained a direct edge from a baseline modifiable exposure to BMI at FU2. Table 5 shows patterns between repeated measurements in the main CCG and the BGs. It can be seen for BMI that in 95 BGs the paths B → FU1 → FU2 or B → FU1 → FU2 ← B were found despite not being contained in the main CCG.

**Table 4:** Directed and partially directed paths between modifiable risk factors at baseline and BMI 6 years later in the original CCG and in 100 Bootstrap graphs (BGs).

	Audio-visual media consumption			
	Partially directed paths from AVM (B) to BMI (FU2)	N	Directed paths from AVM (B) to BMI (FU2)	N
CCG	Shortest path: AVM (B) → AVM (FU1)** − **HOMA (FU1) → HOMA (FU2)** − **BMI (FU2)			
	Number of paths	6		0
BG	Number of BGs with any partially directed path	80		26
	Most frequent path: AVM (B) → AVM (FU1)** − **Well-being (FU1)** − **HOMA (FU1) → HOMA (FU2)** − **BMI (FU2)	11	AVM (B) → Sleep (B) → BMI (B) → BMI (FU1) → BMI (FU2)	4
	3 most frequently visited nodes (based on all paths):			
	YHEI (B)	63%	BMI (FU1)	55%
	AVM (FU1)	62%	HOMA (FU1)	39%
	Sleep (FU1)	55%	YHEI(B)	30%

	Physical activity			
	Partially directed paths from PA (B) to BMI (FU2)	N	Directed paths from PA (B) to BMI (FU2)	N
CCG	Shortest path: PA (B) → YHEI (B) → AVM (FU1) − HOMA (FU1) → HOMA (FU2) − BMI (FU2)			
	Number of paths	13		0
BG	Number of BGs with any partially directed path	80		19
	Most frequent path: PA (B) → PA (FU1) − Well-being (FU1) − HOMA (FU1) → HOMA (FU2) − BMI (FU2)	12	PA (B) → PA (FU1) → Daily family meals (FU2) → Mother’s BMI (FU2) → BMI (FU2)	4
	3 most frequently visited nodes (based on all paths):			
	YHEI (B)	82%	YHEI (B)	61%
	AVM (FU1)	62%	BMI (FU1)	42%
	Well-being (FU1)	59%	Well-being (B)	36%

	Sleep duration			
	Partially directed paths from sleep (B) to BMI (FU2)	N	Directed paths from sleep (B) to BMI (FU2)	N
CCG	Shortest path: Sleep (B) → HOMA (FU1) → HOMA (FU2) − BMI (FU2)			
	Number of paths	2		0
BG	Number of BGs with any partially directed path	75		32
	Most frequent path(s): Sleep (B) → Sleep (FU1) → Sleep (FU2) − Puberty stage (FU2) − zBMI (FU2)	19	Sleep (B) → HOMA (FU1) → BMI (FU2), Sleep (B) → HOMA (FU1) → HOMA (FU2) → BMI (FU2)	8
	3 most frequently visited nodes (based on all paths):			
	AVM (B)	63%	BMI (FU1)	33%
	AVM (FU1)	60%	HOMA (FU1)	32%
	Puberty stage (FU2)	58%	AVM (B)	28%

	Well-being			
	Partially directed paths from well-being (B) to BMI (FU2)	N	Directed paths from well-being (B) to BMI (FU2)	N
CCG	Shortest path: Well-being (B) → YHEI (B) → AVM (FU1) − HOMA (FU1) → HOMA (FU2) − BMI (FU2)			
	Number of paths	6		0
BG	Number of BGs with any partially directed path	81		26
	Most frequent path: Well-being (B) → Well-being (FU1) − HOMA (FU1) → HOMA (FU2) − BMI (FU2)	20	Well-being (B) → HOMA (FU1) → BMI (FU2)	4
	3 most frequently visited nodes (based on all paths):			
	YHEI (B)	78%	HOMA (FU1)	53%
	AVM (FU1)	66%	YHEI (B)	45%
	Sleep (FU1)	61%	BMI(FU1)	45%

	Youth healthy eating index			
	Partially directed paths from YHEI (B) to BMI (FU2)	N	Directed paths from YHEI (B) to BMI (FU2)	N
CCG	Shortest path: YHEI (B) → AVM (FU1) − HOMA (FU1) → HOMA (FU2) − BMI (FU2)			
	Number of paths	5		0
BG	Number of BGs with any partially directed path	84		26
	Most frequent paths: YHEI (B) − Daily family meals (B) − Mother's BMI (B) − BMI (B) → BMI (FU1) → BMI (FU2)	19	YHEI (B) → AVM (B) → AVM (FU1) → Daily family meals (FU2) → Mother's BMI (FU2) → BMI (FU2) YHEI (B) → Daily family meals (B) → Mother's BMI (B) → BMI (B) → BMI (FU1) → BMI (FU2) YHEI (B) → AVM (FU1) → Daily family meals (FU2) → Mother's BMI (FU2) → BMI (FU2)	2
	3 most frequently visited nodes (based on all paths):			
	AVM (FU1)	62%	HOMA (FU1)	32%
	Well-being (FU1)	55%	BMI (FU1)	30%
	Sleep duration (FU1)	54%	AVM (B)	28%

*AVM* audio-visual media consumption, *B* baseline, *BMI* body mass index, *FU1* first follow-up, *FU2* second follow-up, *HOMA* homeostatic model assessment-insulin resistance, *ISCED* highest parental education (International Standard Classification of Education), *PA* physical activity, *Sleep* nocturnal sleep, *YHEI* youth healthy eating index.

**Table 5:** Path patterns between repeated measurements in CCG and Bootstrap graphs.

Pattern	BMI	AVM	PA	Sleep	Well-being	YHEI	HOMA	Daily family meals	Mother’s BMI	Income	ISCED
None	**2**	–	–	2	–	2	0	1	25	0	0
B → FU1	2	2	10	2	–	–	2	**63**	0	0	1
B → FU2	–	1	2	–	1	1	0	2	**45**	0	0
B → FU1, B → FU2	–	1	11	1	2	1	1	34	0	0	0
FU1 → FU2	1	9	1	13	5	1	0	0	28	35	36
B → FU2, FU1 → FU2	–	1	1	1	1	–	0	0	2	5	6
B → FU1 → FU2	82	20	25	37	38	2	**59**	0	0	**57**	**57**
B → FU1 → FU2 ← B	13	**66**	**50**	**44**	**53**	**93**	38	0	0	3	0

*AVM* audio-visual media consumption, *B* baseline, *BMI* body mass index, *FU1* first follow-up, *FU2* second follow-up, *HOMA* homeostatic model assessment-insulin resistance, *ISCED* highest parental education (International Standard Classification of Education), *PA* physical activity, *sleep* nocturnal sleep, *YHEI* youth healthy eating index.

Bold numbers: path included in main CCG.

The BGs contained on average 22 edges more than the CCG in the main analysis. For comparison with this main CCG, we constructed a graph containing the same number of edges based on the most frequent edges; this resulted in the inclusion of all edges that occurred in more than 44 of BGs (see Fig. S6). The (structural) Hamming distance between main CCG and BG44 was 56 (73), indicating that about half of the edges between the two graphs are the same.

### Sensitivity analyses

Using a larger nominal significance level of 10% (CCG MI-0.1) essentially confirmed the core results from the main graph with only few more edges (Table 6, Fig. S3). The CCGs estimated with two alternative methods for missing values (TWD and EM) were with 40 to 50% more edges less sparse than the main graph (cf. Figs. S4, S5), where only 20% of the edges in the main analysis were also found in the TWD graph. This was also reflected by the Hamming distances, which was large with 205 for TWD compared to the main CCG. The structural Hamming distance, which additionally counts directional changes, indicated for the MI-0.1 graph that the increase of the nominal level resulted in some undirected edges being directed (e.g., well-being (FU2) → YHEI (FU2)), or vice versa, and others to be re-directed (e.g., the edge between PA (B) and YHEI (B)).

**Table 6:** Characteristics of the discovered graph without singletons.

Characteristics	Main	MI-0.1	TWD	EM	Avg.BG	BG44	BG75
Number of selected edges	104	113	139	157	126	104	46
Number of undirected edges	12	13	14	0	12	3	0
Avg. node degree	4.8	4.9	6.0	6.2	5.4	4.2	1.8
Max. node degree	10^a^	11^b^	13^c^	24^d^	12	9^e^	6^e^
Avg. shortest path length	2.8	2.7	2.4	2.4	2.7	2.2	1.4
Longest shortest path	9^g^	8^h^	7^i^	7^j^	8	6^k^	5^l^
Hamming distance^64^	–	19	205	117	88	56	70
Structural Hamming distance^65^	–	34	214	131	104	73	86
Mean edge uncertainty^54^	–	–	–	–	10.5	4.4	0.8

*Avg.BG* occurs on average in each BootG, *BGx* summarized bootstrap graph with edges that occurred at least × times in 100 bootstrap replications, *EM* structural EM algorithm, *main* multiple imputation with nominal level of 0.05, *MI-0.1* multiple imputation with nominal level of 0.1, *MEU* mean edge uncertainty^54^, *TWD* test-wise deletion.

^a^Region, AVM (FU1), well-being (B).

^b^AVM (FU1), well-being (B).

^c^Migrant.

^d^Region.

^e^School (B).

^g^Age (FU1) > School (FU1) > Daily family meals (FU1) > YHEI (FU1) > Well-being (FU1) > AVM (FU1) > HOMA (FU1) > HOMA (FU2) > BMI (FU2).

^h^Age (FU1) > School (FU1) > Daily family meals (FU1) > YHEI (FU1) > AVM (FU1) > HOMA (FU1) > HOMA (FU2) > BMI (FU2).

^i^Weeks of pregnancy (EL) > Daily family meals (B) > Sleep (B) > AVM (B) > Well-being (B) > HOMA (B) > BMI (FU2).

^j^Smoking during pregnancy (EL) > Weeks of pregnancy (EL) > Birthweight (EL) > Mother`s BMI (B) > AVM (B) > Well-being (B) > Well-being (FU1).

^k^Age (B) > School (B) > Well-being (B) > YHEI (B) > PA (B) > PA (FU1).

^l^Age (B) > School (B) > Well-being (B) > Well-being (FU1) > Well-being (FU2).

## Discussion

The estimated CCG suggested rather sparse causal relationships between various variables around childhood obesity, with dependencies of repeated measures across time being the strongest and most stable as one might expect. All the individually modifiable risk factors diet, PA, sleep duration, subjective well-being and audio-visual media consumption at baseline were stably found to be possible indirect, but not direct, causes of BMI 6 years later, mostly via the HOMA index which was closely linked to BMI. Associations between media exposure^36–39^, sleep^40–42^, PA^40^, diet^40^, well-being^41^ and insulin resistance measured by HOMA were previously found by others and in the IDEFICS/I.Family cohort, partly in smaller subsets and using different variables such as objective accelerometer-based measurements of PA^43–45^. Insulin resistance is strongly associated with obesity, which is reflected by an undirected edge in the CCG. Excess adipose tissue is a known risk factor for insulin resistance; however, normal-weight children may also be affected^46^. From the early life factors, birthweight was a (possible) ancestor of BMI (B, FU1, FU2) and formula milk feeding for BMI (FU1, FU2). High birth weight is known to be associated with childhood obesity^47^; and a recent systematic review describes that there is moderate evidence that breast milk consumption reduces the risk of overweight and obesity at age 2 years and older^48^.

Overall, our results suggested that early life interventions targeting health behaviours of the child will have some, but only indirect effects on BMI^49^. Hence, cultural, perinatal and familial variables are potentially more immediate causal influences on obesity. Based on the selected CCG, we might therefore hypothesise that early life interventions alone may be insufficient to prevent childhood obesity. Indeed, Börnhorst et al.^49^ found that even *sustained* (over 13 years) and *joint* hypothetical interventions on multiple modifiable behaviours only reduced the risk of obesity in children from 31 to 25%. Thus, our finding is compatible with the view that the causal structure governing childhood health behaviours and outcomes should be considered from a complex adaptive system's perspective^50–52^. Lee et al.^50^ emphasize that obesity is shaped by multiple factors which act at different scales such as individual behaviour and physiology, but also genetics, social dynamics, the built environment, and societal forces. As a way forward, Maitland et al.^53^, for example, describe the practical implementation of a “whole of systems” approach.

Using sensitivity analyses we investigated the robustness of the CCG regarding the handling of missing values and used bootstrap samples to assess the stability of learned graph structures. The method for handling missing values is not negligible as more complex and quite different graphs were estimated using TWD or the EM-algorithm instead of MI. Moreover, it was noticeable that the TWD graph, unlike the CCG, was not able to detect edges between repeated measurements. Witte et al.^27^ showed that TWD can fail in recovering certain causal structures regardless of the underlying missingness mechanism (MCAR, MAR or MNAR). Further, MI was usually more efficient than TWD, although datasets including variables with mixed measurement scales were more problematic.

We used bootstrap resamples to account for the uncertainty in the selection of the CCG^54–56^. In interpreting the results, it has to be kept in mind that the BGs tended to have more edges than the main CCG, due to spurious dependences induced by sampling with replacement from the given data^56,57^. We therefore considered the BGs purely as a measure of the stability rather than, say, for estimating edge probabilities. Thus, edge and path frequencies indicate the stability of presence and absence of certain graph structures. While about a third of the learned edges in the main analysis were quite stable, we also found that half of the edges were rather unstable. Similarly, we found that the existence of some paths from early modifiable risk factors to later BMI was quite stable, but the actual paths themselves were very variable, i.e. a particular path may not be selected in more than 20% of BGs. In contrast, the absence of direct links from early modifiable risk factors to later BMI was very stable as these occurred in no BGs. This can be interpreted as the absence of direct causal influences even when the assumption of causal sufficiency is violated.

The main analysis was able to find the expected paths for repeated measurements of HOMA and all modifiable risk factors, but not for BMI, and only partly for daily family meals and mother’s BMI. The BGs runs revealed that missing edges between the repeated measurements of BMI are very rare. The CCG is therefore difficult to explain in this respect. In contrast, the learned CCG suggests the plausible relationship that BMI is conditionally independent of modifiable risk factors given the child's insulin resistance status (HOMA).

The instabilities that we found through the bootstrap analysis might partly be explained by the rather low sample size for the perhaps rather weak associations, the extra uncertainty due to the high proportion of missing values, and the large intervals between follow-ups. Especially the confidence in specific paths might be rather low which is critical. A greater stability would, for instance, be desirable for subsequent analyses that use a learned causal graph to determine adjustment sets to estimate causal effects^8^. Some graphical rules for identifying adjustment sets just take the adjacent nodes of the exposure into account and others require also the mediators between exposure and outcome, for which reliable knowledge on causal paths is required^58,59^.

Recently, Peterson, Osler & Ekstrom^14^ also proposed an extension of the PC-algorithm to include temporal information for inferring a graph from observational data. However, our extensions of the PC-algorithm allows the first application of causal discovery to real-world cohort data accounting jointly for missing values, mixed discrete and continuous variables, and background knowledge such as time-ordering. The required theory and software have only recently been developed^11,27^.

The IDEFICS/I.Family cohort provides a rich source of phenotypes capturing different dimensions of dietary and lifestyle related health aspects repeatedly measured over the early life course. However, a challenge was the choice of variables included in the analysis; these needed to be sufficiently different (i.e. not measuring the same underlying construct) to find meaningful dependencies between the different dimensions of obesity. The further sensitivity analyses (see web page) showed that different choices yielded slightly different selected CCGs, but the overall message remained the same: adolescents’ BMI was not directly affected by earlier behavioural variables, but had indirect, potentially causal, links through AVM (FU1) and HOMA (FU1, FU2).

Further general sources of bias with observational data could also affect our results, such as reporting or selection bias. However, all participating countries adhered to a harmonised protocol and to quality control procedures ensuring high data quality.

## Conclusion

Causal graphs represent causal relationships between variables. An extended version of the PC algorithm now allows learning causal graphs from tiered data including missing values. Such a causal graph discovery analysis was performed on the IDEFICS/I.Family cohort investigating (causal) dependencies underlying childhood and adolescent obesity in 2 to 16-year-old Europeans.

The resulting CCG suggested that cultural, perinatal and familial factors and insulin resistance (HOMA-IR) potentially played a more immediate causal role than individually modifiable risk factors which had stable but only indirect relations with adolescents’ BMI.

### Supplementary Information

Supplementary Information.

## Data Availability

All CCGs are available as interactive graphs at https://bips-hb.github.io/ccg-childhood-obesity/. The R analysis code is available at https://github.com/bips-hb/ccg-childhood-obesity. All data analyzed within the paper were obtained from the IDEFICS/I.Family cohort and is available from the I.Family consortium (http://www.ifamilystudy.eu) on reasonable request.
